# The Protective Effect of Walnut Oligopeptides against Indomethacin-Induced Gastric Ulcer in Rats

**DOI:** 10.3390/nu15071675

**Published:** 2023-03-30

**Authors:** Rui Liu, Na Zhu, Yuntao Hao, Xinran Liu, Jiawei Kang, Ruixue Mao, Xiaochen Yu, Yong Li

**Affiliations:** Department of Nutrition and Food Hygiene, School of Public Health, Peking University, Beijing 100191, China; liuruipku@bjmu.edu.cn (R.L.);

**Keywords:** walnut oligopeptides, gastric ulcer, indomethacin, prostaglandin E2, arachidonic acid metabolism

## Abstract

The aim of this study was to investigate the potential protective effects of walnut oligopeptides (WOPs) on indomethacin-induced gastric ulcers in rats. The rats were divided into the following groups: normal group, model group, omeprazole group (0.02 g/kg), and WOPs groups (0.22, 0.44, and 0.88 g/kg, respectively). After receiving gavage once per day for 30 consecutive days, the rats were injected intraperitoneally with indomethacin 48 mg/kg to induce gastric ulcers. Then, the serum inflammatory cytokines and gastric prostaglandin E2 (PGE2), oxidative stress-related indicators, and the RNA expression of COX-1 and COX-2 were measured. The results revealed that WOPs confer significant gastroprotection on gastric ulcers caused by indomethacin, regulating inflammatory cytokines, oxidative stress, and prostaglandins synthesis, and enhancing the expression of COX-1 and COX-2 in gastric tissue, thus exerting its protective effect on gastric mucosa. The gastroprotective mechanism may be related to the involvement of the arachidonic acid metabolism and upregulation of tryptophan, phenylalanine, tyrosine, and alpha-Linolenic acid metabolism synthesis in vivo.

## 1. Introduction

Nonsteroidal anti-inflammatory drugs (NSAIDs) are the most commonly used medications in the world. This category includes aspirin, indomethacin, diclofenac, and ibuprofen, which are widely used for arthritis, inflammation, pain, fever treatment, and cardiovascular protection due to their powerful anti-inflammatory, anti-pyrogenic, and anti-thrombotic properties [1]. However, NSAIDs have various adverse effects on the gastrointestinal tract, liver, nervous, urinary tract, blood, and cardiovascular system, especially those occurring in the stomach. These effects can lead to issues, such as gastric mucosal erosion, ulcer, hemorrhage, and perforation [2]. In recent decades, with the increasing clinical application range and dosage, the incidence rate of NSAIDs-related gastric ulcer is also on the rise [3]. The annual incidence rate of NSAID-related upper gastrointestinal clinical events is 2.7~4.5%, and among patients taking NSAIDs, the incidence of gastric ulcers is about four times that of duodenal ulcers [4,5]. The pathophysiology of a gastric ulcer induced by NSAIDs has mostly been ascribed to its inhibition of both isoforms of cyclooxygenases (COX-1 and -2) expression via interference with the metabolism of arachidonic acid (AA), reducing the synthesis of gastric mucosal protective factor prostaglandins (PGs) [5,6].

According to its pathogenic mechanism, various drugs, including COX-2 selective inhibitors (e.g., celecoxib), acid inhibitors (e.g., proton pump inhibitor (PPI)), mucosal protective agents (e.g., bismuth and teprenone), and prostaglandin derivatives (e.g., misoprostol), were adopted to prevent or treat NSAIDs-induced gastric ulcers [5,7]. However, these treatments have certain limitations and varying degrees of side effects: misoprostol can cause diarrhea, abdominal pain, and cramping, which can limit its use, and sucralfate has limited effectiveness in preventing gastrointestinal complications and can interfere with the absorption of other medications [8]. Additionally, the long-term use of PPI has been associated with an increased risk of infections, dysbiosis, hypomagnesemia, and osteoporosis, while chronic PPI use may interfere with the absorption of other drugs, such as clopidogrel, which is used to prevent blood clots [8]. Moreover, Boghossian, T. A. et al. found that even with on-demand PPI use rather than once-daily PPI treatment, a significant proportion of people were reluctant to continue taking these medications due to the recovery of gastrointestinal symptoms [9]. Therefore, it is of great significance to find new agents with few adverse effects to protect against gastric injury induced by NSAIDs. In recent years, bioactive peptides have attracted increasing attention due to their multiple physiological functions, such as immunomodulatory, antimicrobial, hormone regulation, antioxidant properties, etc. [10]. Studies have shown that a variety of bioactive peptides are able to protect against gastric mucosal injury caused by alcohol through mechanisms mainly related to the inhibition of alcohol-induced oxidative stress and the accumulation of inflammatory factors, indicating that bioactive peptides have huge promise in protecting against gastrointestinal injury and are beneficial to health [11,12,13]. Walnut oligopeptides (WOPs), which have characteristics such as a low molecular weight, high absorption, bioavailability, and safety, are extracted from walnut protein by using biological enzyme hydrolysis technology. Studies have found that WOPs have numerous potential physiological functions, including anti-fatigue [14], improved anti-oxidation capacity [15], improved aging-related learning and memory impairment [16], and the inhibition of cell apoptosis of spleen tissue induced by ionizing radiation in mice [17]. However, the effect of WOPs on NSAID-related gastric ulcers is rarely reported. In addition, among the commonly used NSAIDs, including aspirin, indomethacin nabumetone, and ibuprofen, indomethacin is often chosen as the inducer of gastrointestinal injury due to its higher potential for inducing serious gastrointestinal toxicity compared to other NSAIDs [18]. Therefore, this study was designed to investigate the beneficial effects of WOPs on indomethacin-induced gastric ulcers and the possible mechanism involved.

## 2. Materials and Methods

### 2.1. Preparation and Identification of WOPs

The WOPs sample was a mixture of small molecular peptides derived from enzymatic hydrolysis of walnut (*Juglans Regia* L.) protein, provided by Jilin Taigu Biological Engineering Co., Ltd. (Jilin, China). Briefly, walnut protein was hydrolyzed with various proteases after cleaning, mincing, and homogenization. Next, nanofiltration, freeze concentration, decolorization, purification, and spray drying were carried out to extract peptide powder. Then, the oligopeptides sample was purified via high-performance liquid chromatography (HPLC, Agilent, CA, USA) using a Phenomenex C18 column (10 mm × 250 mm). The molecular weight distribution was measured by LDI-1700 matrix-assisted laser desorption ionization time-of-flight mass spectrometry (MALDI-TOF-MS, Liner Scientific Inc., Reno, NV, USA), and the free amino acids amount of WOPs sample was analyzed by the automatic amino acid analyzer (Hitachi, Tokyo, Japan). The results of WOPs identification showed that the content of oligopeptides with molecular weights between 180 and 1000Da was 86.5%; the amino acid composition has been reported in detail in our previous studies [14,15,17].

### 2.2. Chemicals and Reagents

Indomethacin was purchased from Sigma Chemical Co. (St. Louis, MO, USA). Omeprazole was manufactured by Hunan Dino Pharmaceutical Limited by Share Ltd. (Hunan, China). The Enzyme-linked immunosorbent assay (ELISA) kits including prostaglandin E2 (PGE2), interleukin (IL)-1β, tumor necrosis factor α (TNF-α) and interleukin (IL)-17A were purchased from Capital Bio Corporation (Beijing, China). The glutathione (GSH), superoxide dismutase (SOD), malondialdehyde (MDA), nitric oxide (NO), and myeloperoxidase (MPO) kits were manufactured by Nanjing Jiancheng Bioengineering Institute (Nanjing, China).

### 2.3. Animals and Experimental Design

A total of 60 male Sprague Dawley rats weighing 180-220 g were purchased from the Department of Laboratory Animal Science, Peking University. The rats were housed in a barrier-level animal room with a temperature range of 22 ± 2 °C, relative humidity of 50–60%, and day/night alternation time of 12 h:12 h. The experiment was reviewed and approved by the Ethics Committee of Peking University Health Science Center (LA2018234), and all animals were treated according to the Principles of Laboratory Animal Care and the guidelines of the Peking University Animal Research Committee.

After one week of adaptive feeding, based on the body weight, SD rats were randomly assigned to six groups, with ten rats per group. The normal and model groups received (i.g.) distilled water. The omeprazole group received (i.g.) omeprazole (0.02 g/kg body weight, dissolved in distilled water) as a reference of anti-ulcer drug [19,20]. The WOPs groups received (i.g.) various doses of WOPs (0.22, 0.44, and 0.88 g/kg body weight, dissolved in distilled water). The dose of WOPs was selected according to our previous studies and preliminary experiment [14,17]. Daily intragastric gavage intervention with a volume of 1 mL/100 g was performed for 30 days, and the body weight and food intake of rats was recorded once a week using electronic scales.

### 2.4. Indomethacin-Induced Gastric Ulcer

On day 30, all rats were fasted for 24 h but drank water freely. Then, the rats were injected once intraperitoneally (i.p.) with freshly prepared indomethacin at a dose of 48 mg/kg body weight in 1% tewwn80 to induce ulceration, while rats in the normal group received only vehicle (no indomethacin) in an identical manner [21,22,23]. Rats were sacrificed 1 h post-indomethacin injection, blood samples were obtained via femoral artery, and then serum was obtained at 3000 rpm centrifugation for 15 min. The gastric tissue of each rat was immediately removed for further analysis.

### 2.5. Evaluation of Gastric Mucosal Injury

The gastric tissue was opened along the greater curvature, and then rinsed slightly with ice-cold 0.9% sodium chloride solution. The mucosal damage was measured and expressed as the gastric ulcer index (GUI), which was scored as described previously by Guth with some modifications: 0 point, no damage; 1 point, spot erosion or erosion length <1 mm; 2 points, erosion length between 1 and 2 mm; 3 points, erosion length between 2 and 3 mm; 4 points, erosion length between 3 and 4 mm. The erosion length longer than 4 mm was segmented scored. The erosion width was larger than 2 mm, the score was doubled [24,25].

### 2.6. Transmission Electron Micrograph Analysis of Gastric Injury

The standardized specimens of gastric tissues (three per group) were fixed into glutaraldehyde at 4 °C for 2 h, fixed with 1% osmium tetroxide for 1.5 h, and stained with uranium dioxide acetate at room temperature for 1 h. A 15 min gradient dehydration process was performed with various concentrations of ethanol. Then, samples were immersed in epoxy resin and acetone for 2h, embedded, and polymerized with epoxy resin. The slices were made by ultra-thin microtome (Leica EM UC6) and dyed with uranium acetate and lead citrate. Investigation was carried out using a JEM-100CXII transmission electron microscope (JEOL Ltd., Tokyo, Japan) and representative images were presented.

### 2.7. Biochemical Assay of Serum and Gastric Tissues

After evaluation of the GUI, serum TNF-α, IL-1β, IL-17A activities, and gastric tissue PGE2, SOD, NO, GSH, MPO, and MDA levels were detected according to the kits instructions, as listed in Section 2.2.

### 2.8. Expression of COX-1 and COX-2 mRNA by RT-PCR

Total RNA was extracted from 50 mg of rat gastric tissue (6 per group) using Trigol reagent (Genview, Beijing, China). RNA was used to prepare cDNA using the Toyobo reverse transcription kit. An equal amount of cDNA was used for PCR amplification by the ABI PRISM 7500 real-time PCR analysis system, and specific forward and reverse primers for COX-1, COX-2, and internal standard GAPDH were adopted. The primer preparation sequences were as follows: for GAPDH, Forward 5′- GTATCGGACGCCTGGTTAC -3′ and Reverse 5′- CTGTGCCGTTGAACTTGCC-3′; for COX-1, Forward 5′- GGAGGTGTTTGGGTTGCT -3′ and Reverse 5′- CCTATAAGGATGAGGCGAGT-3′; for COX-2, Forward 5′- GGGTAATCCCATCTGTTCTC -3′ and Reverse 5′- ACTTGCGTTGATGGTGGC -3′. The cycling conditions were pre-denaturation at 94 °C for 2 min, denaturing at 94 °C for 30 s, annealing at 58 °C for 30 s, and extension at 72 °C for 30 s for each primer set, and then a final extension at 72 °C for 10 min. Next, the products were dissolved in a 2% agarose gel and electrophoresed at a voltage of 5V/cm for 30 min, and the electrophoresis bands of the PCR products were documented under a gel imaging system (LiuYi Biotechnology Co., Ltd., Beijing, China). Finally, the relative amount of mRNA was normalized against GAPDH levels, and the fold change for each mRNA was counted according to the comparative cycle threshold (△△Ct) method.

### 2.9. Untargeted Metabolomics

The plasma samples from the model group, omeprazole group, and WOPs-HG (0.88 g/kg body weight) group were collected (6 per group) for untargeted metabolomics analysis. The metabolites were extracted from the samples according to the experimental procedures, and Waters 2D UPLC (waters, USA) tandem with high-resolution mass spectrometer Q Exactive HF (Thermo Fisher Scientific, USA) was used for metabolite separation and detection. The column used was a BEH C18 column (1.7 μm 2.1 ∗ 100 mm, Waters, USA). The positive ionization mode mobile phase was aqueous solution containing 0.1% formic acid in water (liquid A) and 100% methanol containing 0.1% formic acid (liquid B), and the negative ionization mode mobile phase was 10 mM ammonia formate in water (liquid A) and 95% methanol containing 10 mM ammonia formate (liquid B). (Liquid A) and 95% methanol containing 10 mM formic acid ammonia (Liquid B). Primary and secondary mass spectrometry data acquisition was performed using a Q Exactive HF mass spectrometer (Thermo Fisher Scientific, Waltham, USA). The scan mass-to-nucleus ratio range was 70~1050, the primary resolution was 120,000, the AGC was 3e6, and the maximum injection time was 100 ms. Top3 were selected for fragmentation according to the parent ion intensity, and secondary information was collected with a secondary resolution of 30,000, an AGC of 1 × 10^5^ a maximum injection time of 50 ms, and the stepped nce set to 20, 40, 60 eV. Next, the data obtained by LC-MS/MS were processed using Compound Discoverer 3.0 (Thermo Fisher Scientific, USA) software. The original multivariate data were analyzed according to principal component analysis (PCA) to reduce the dimensionality of the observed variables in the data set. The differential metabolites were screened by combined analysis of Partial Least Squares Method-Discriminant Analysis (PLS-DA), values of Variable Importance in Projection (VIP), the fold change obtained from univariate analysis, and Student’s *t*-test. Significant differences in metabolites were defined as metabolites with VIP>1, fold change ≥1.2 or ≤0.83, and *p* < 0.05 (Student’s *t*-test). Furthermore, pathway enrichment analysis was performed using the KEGG (http://www.genome.jp/kegg/, accessed on 18 October 2022) and MetaboAnalyst (http://www.metaboanalyst.ca/, accessed on 18 October 2022) databases.

### 2.10. Statistical Analysis

Data are presented as mean ± SEM for bar and line graphs. SPSS24.0 software was used for statistical analysis. Comparison between multiple groups was statistically analyzed using the one-way analysis of variance (ANOVA) test with least significant difference (LSD) methods. A value of *p* < 0.05 was considered a statistically significant difference.

## 3. Results

### 3.1. Body Weight and Food Intake

Over the experimental period, the weight gain of the rats in each group reflected normal growth. However, there was no significant change in the body weight, food intake, or food utilization between the groups (*p* > 0.05) (Table 1).

### 3.2. Effect of WOPs on Macroscopic Gastric Mucosal Injury

**Figure 1:**
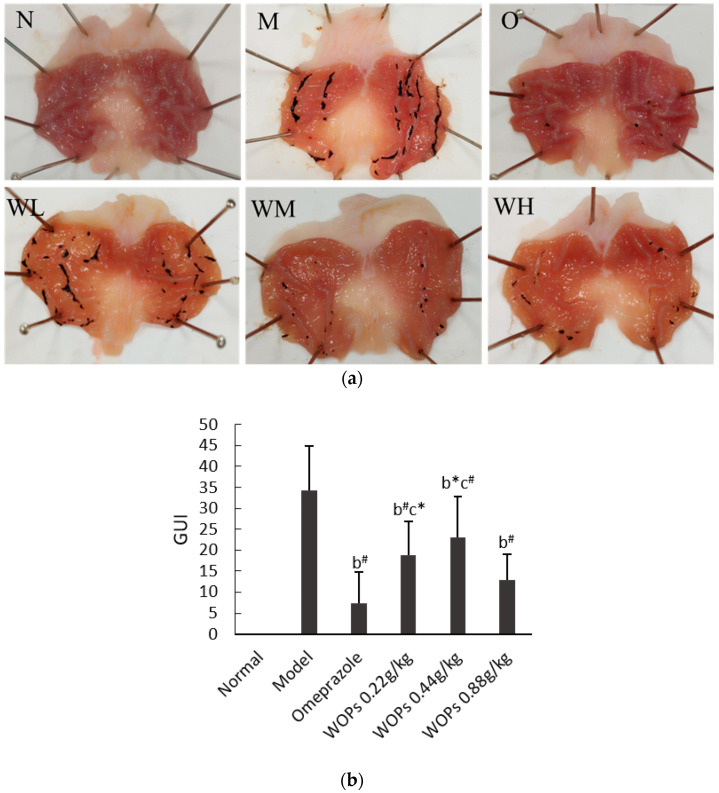
Effect of WOPs on gross appearances of gastric mucosal (**a**) and gastric ulcer index (**b**) in indomethacin-induced gastric ulcer in rats. N, normal group; M, model control group; O, omeprazole group (0.02 g/kg); WL, WOPs 0.22 g/kg; WM, WOPs 0.44 g/kg; WH, WOPs 0.88 g/kg. Significance was represented as * *p* < 0.05, # *p* < 0.01; b represents comparison with the model group and c refers to comparison with the omeprazole group; 10 per group. GUI, gastric ulcer index; WOPs, walnut oligopeptides.

As shown in Figure 1a, no macroscopic lesion was observed in the normal group (group N), whereas the rats in the model group treated with indomethacin at a dose of 48 mg/kg exhibited serious gastric mucosal injury in the form of linear or striped bleeding bands. However, in the omeprazole group, the observed gastric mucosal damage was the lightest among all the groups, and most of the rats had no inflammatory reaction or injury (group O). The three WOPs pretreatment groups (WL, WM, and WH groups, respectively) showed medium to slight gastric mucosal damage compared to the model group, and the WOPs-HG (0.88 g/kg body weight) group exhibited the lightest gastric mucosa injury.

The outcomes shown in Figure 1b highlight that the GUI was significantly increased in the model group compared with the omeprazole group and the three groups of WOPs (*p* < 0.05 or *p* < 0.01). While the GUI of the omeprazole group was obviously lower than that of the WOPs-LG and WOPs-MG groups (*p* < 0.05 or *p* < 0.01), no statistical difference was observed between the omeprazole and WOPs-HG groups (*p* > 0.05).

### 3.3. Transmission Electron Micrograph Images of Gastric Injuries

**Figure 2:**
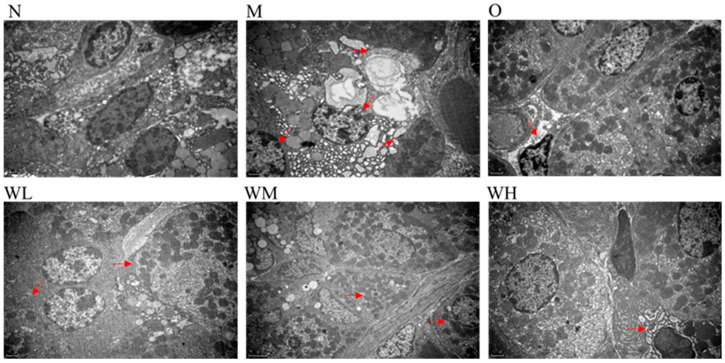
Transmission electron micrograph images of gastric injuries (three per group). The red arrows represent major changes in the organelles. N, normal group; M, model control group; O, omeprazole group (0.02 g/kg); WL, WOPs 0.22 g/kg; WM, WOPs 0.44 g/kg; WH, WOPs 0.88 g/kg. WOPs, walnut oligopeptides.

The normal group showed a clear cell structure with round nuclei and a basically uniform distribution of chromatin in the nucleus, the mitochondria were abundant in the cytoplasm, and there were many stacked rough endoplasmic reticulum in the cells (N in Figure 2). However, the structure of the gastric mucosa cells in the model control group was disordered, the chromatin was sparse, the mitochondria were swollen, the lysosomes increased, and the rough endoplasmic reticulum decreased and vesicled (M in Figure 2). In the omeprazole (O in Figure 2) and WOPs pretreated groups (WL–WH in Figure 2), the transmission electron micrographs revealed that with the increase in the intervention dose, the cell structure of the gastric mucosa gradually approached the normal state, exhibiting that the degree of cell swelling decreased, the nucleus morphology became regular, the chromatin in the nucleus was uniform, and the rough endoplasmic reticulum was abundant.

### 3.4. Effect of WOPs on the PGE2 Contents

**Figure 3:**
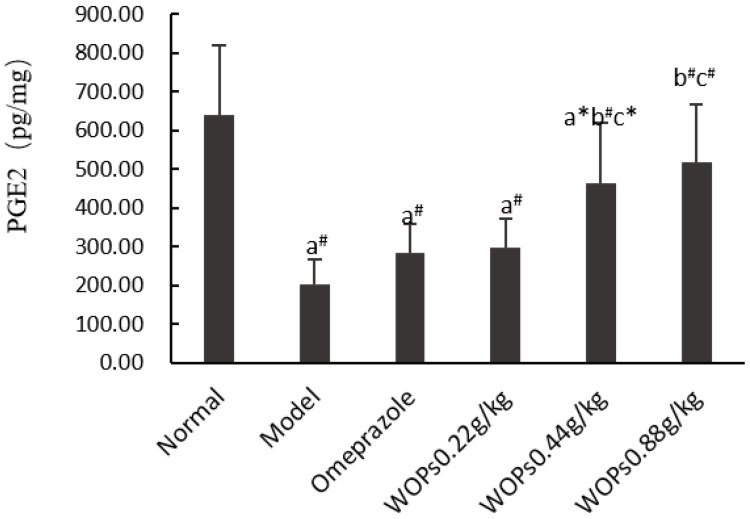
Effects of WOPs on PGE2 synthesis in gastric tissue of indomethacin-induced rats. Significance was represented as * *p* < 0.05, # *p* < 0.01; a represents comparison with the normal group, b refers to comparison with the model group, and c refers to comparison with the omeprazole group; 10 per group. PGE2—prostaglandin E2; WOPs—walnut oligopeptides.

The gastric prostaglandin E2 (PGE2) levels were obviously reduced in the model, omeprazole, and WOPs 0.22 and 0.44 g/kg groups compared to the normal group of rats (*p* < 0.01) (Figure 3). However, in the medium- and high-dose groups of WOPs (0.44 and 0.88 g/kg), the PGE2 levels were remarkably elevated compared to the model and omeprazole groups (*p* < 0.05 or *p* < 0.01). The findings indicated that pretreatment with WOPs could enhance the PGE2 deficiency caused by indomethacin in gastric mucosa.

### 3.5. Effects of WOPs on the NO and MPO Levels

**Figure 4:**
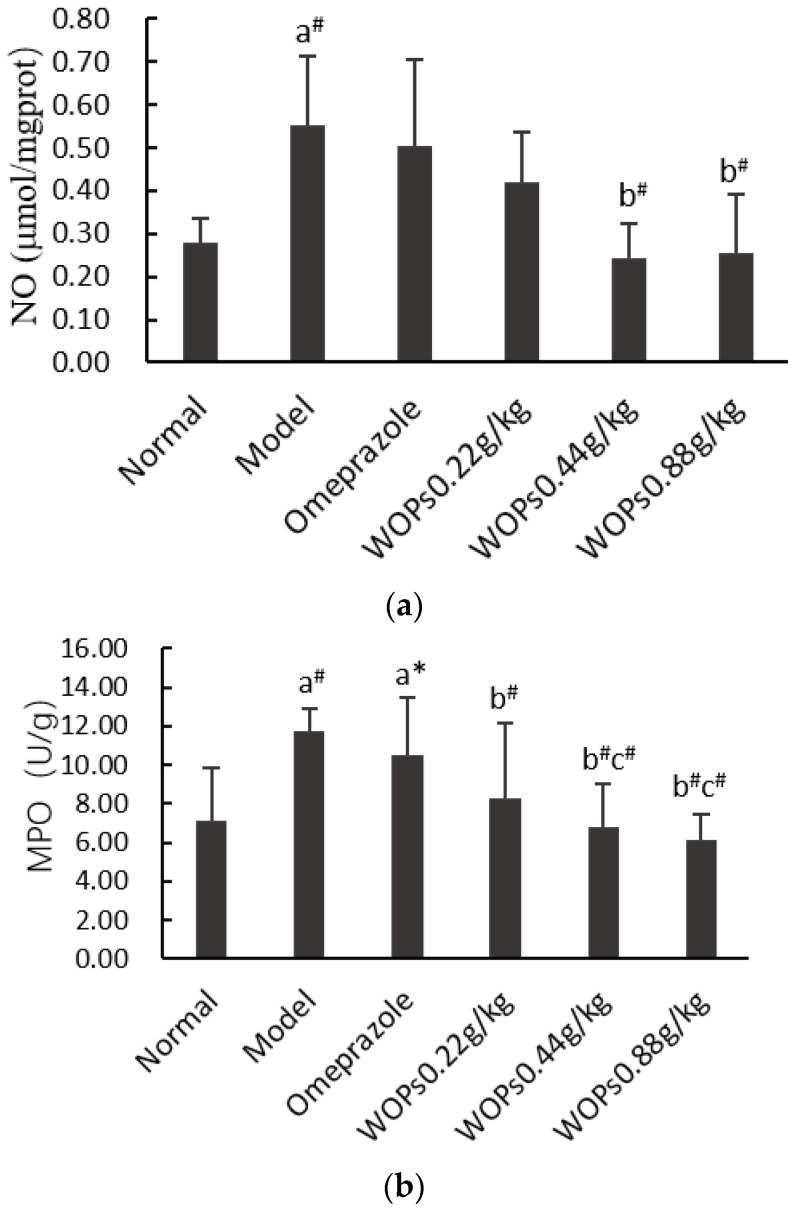
Effects of WOPs on gastric NO (**a**) and MPO (**b**) levels in rats. Significance was represented as * *p* < 0.05, # *p* < 0.01; a—compared with normal group, b—compared with model group, c—compared to the omeprazole group; 10 per group. NO—nitric oxide; MPO—myeloperoxidase; WOPs—walnut oligopeptides.

The contents of nitric oxide (NO) (Figure 4a) and myeloperoxidase (MPO) (Figure 4b) were remarkably elevated in the model group compared to the normal group (*p* < 0.01). In contrast, in the WOPs-treated group, the levels of NO and MPO were markedly declined compared with the model group (*p* < 0.01), except for the NO level in the WOPs 0.22 g/kg group. The MPO level in the WOPs pretreatment groups (0.44 and 0.88 g/kg) was markedly decreased in comparison with that of the omeprazole group (*p* < 0.01), but the NO levels were not significantly different (*p* > 0.05). These findings indicated that the WOPs pretreatment could prevent indomethacin-induced gastric ulcers to a certain extent.

### 3.6. Effect of WOPs on Indomethacin-Induced Oxidative Stress

**Figure 5:**
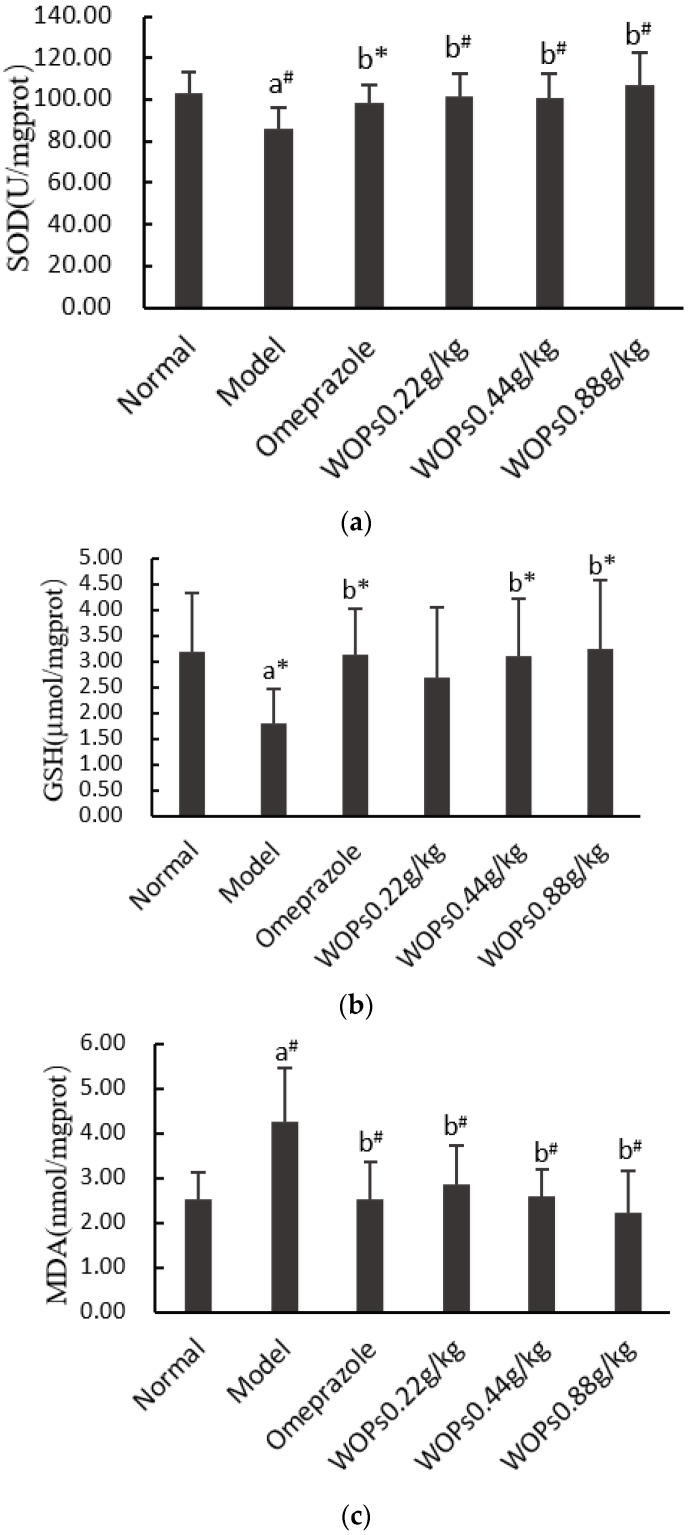
Effects of WOPs on SOD (**a**), GSH (**b**), MDA (**c**) levels in indomethacin-treated rats. Significance was represented as * *p* < 0.05, # *p* < 0.01; a—compared with normal group, b—compared with model group; 10 per group. SOD—superoxide dismutase; GSH—reduced glutathione; MDA—malondialdehyde; WOPs—walnut oligopeptides.

As presented in Figure 5, indomethacin triggered a decrease in superoxide dismutase (SOD) and reduced the glutathione (GSH) levels and there was an increase in the malondialdehyde (MDA) levels in the model group compared with the normal group (*p* < 0.01, *p* < 0.05, and *p* < 0.01, respectively). Compared with the model group, the levels of SOD and GSH were markedly elevated, and the MDA level significantly decreased when the rats were orally administrated omeprazole and WOPs (0.22, 0.44, and 0.88 g/kg) (*p* < 0.05 or *p* < 0.01), except for the GSH content in the WOPs 0.22g/kg group. However, there was no obvious difference between the omeprazole and WOPs groups (*p* > 0.05).

### 3.7. Effect of WOPs on the Release of Inflammatory Cytokines

**Figure 6:**
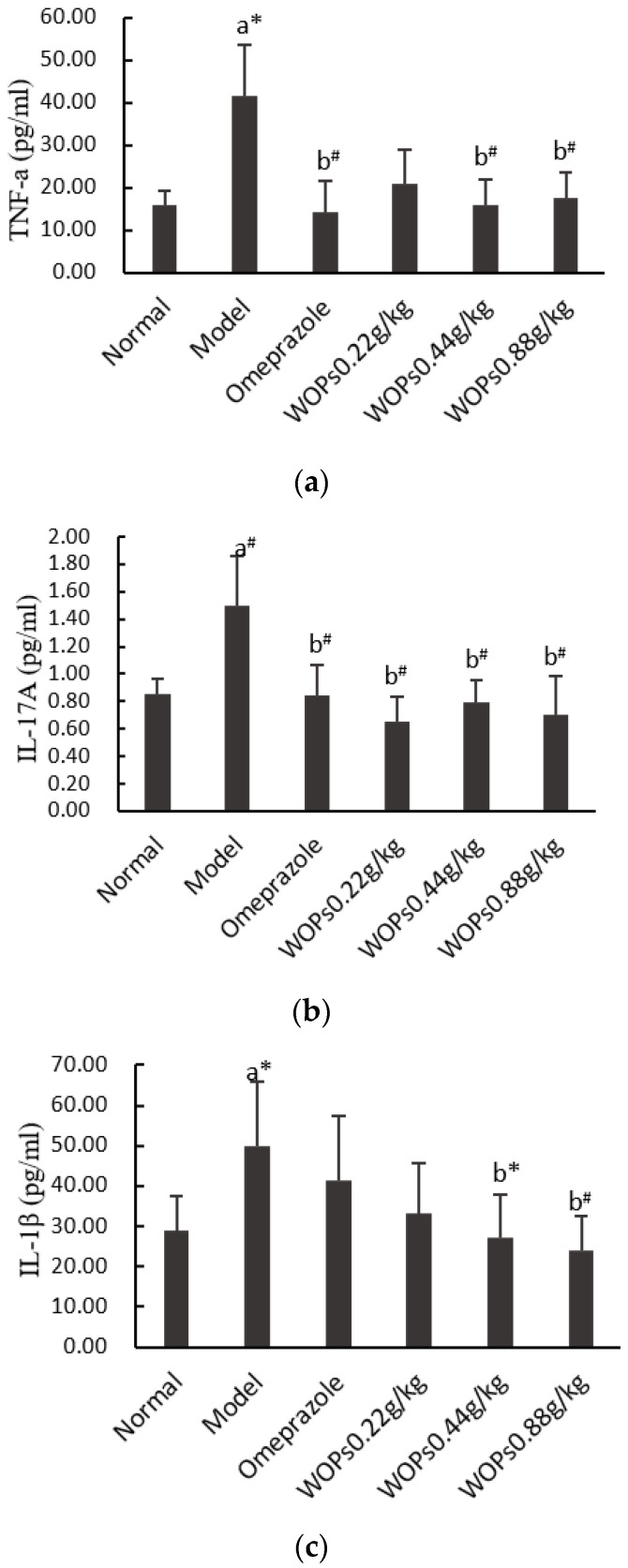
Effects of WOPs on serum TNF-α (**a**), IL-17A (**b**), IL-1β (**c**) levels in indomethacin-treated rats. Significance was represented as * *p* < 0.05, # *p* < 0.01; a—compared with normal group, b—compared with model group; 10 per group. Tumor necrosis factor α—TNF-α; interleukin (IL)-17A—IL-17A; interleukin (IL)-1β—IL-1β; WOPs—walnut oligopeptides.

As shown in Figure 6, the TNF-α, IL-17A, and IL-1β levels in the model group rats were markedly greater than those in the normal group rats (*p* < 0.05, *p* < 0.01, and *p* < 0.05, respectively), which indicated that indomethacin caused an overreaction of the inflammatory cells. The TNF-α, IL-17A, and IL-1β levels were obviously decreased in the WOPs pretreatment group compared with the model group (*p* < 0.05 or *p* < 0.01), except for the levels of TNF-α and IL-1β in the WOPs 0.22g/kg group.

### 3.8. Effect of WOPs on mRNA Expression of COX-1 and COX-2 in Gastric Tissue

**Figure 7:**
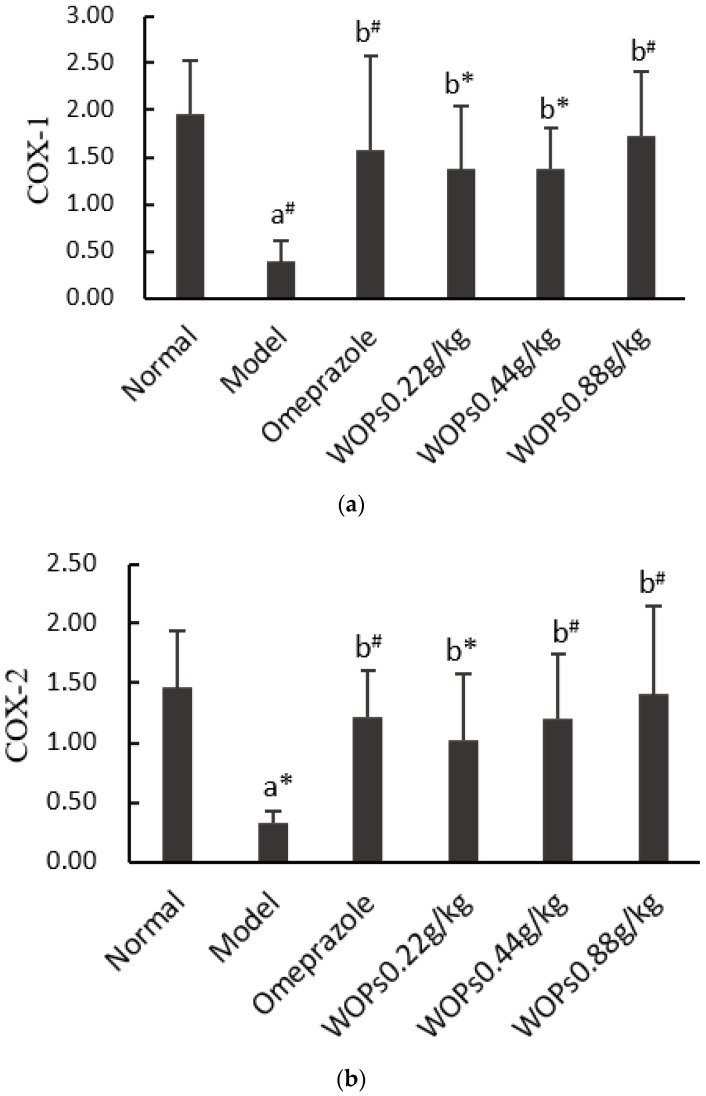
Effects of WOPs on the RNA expression of COX-1 (**a**) and COX-2 (**b**) in gastric tissue. Significance was represented as * *p* < 0.05, # *p* < 0.01; a—comparison with the normal group, b—comparison with the model group; 6 per group. COX—cyclooxygenase; WOPs—walnut oligopeptides.

Both COX-1 and COX-2 are the core enzymes of the arachidonic acid metabolism, which are considered to be the primary regulators of NSAIDs-induced gastric ulcers. The expression levels of COX-1 (Figure 7a) and COX-2 (Figure 7b) were markedly suppressed in the model group compared to the normal group (*p* < 0.01 and *p* < 0.05, respectively). In contrast, the mRNA expression of COX-1 and COX-2 was significantly higher in the omeprazole and three WOPs pretreatment (0.22, 0.44, and 0.88 g/kg) groups than in the model group (*p* < 0.05 or *p* < 0.01).

### 3.9. Effect of WOPs on Plasma Metabolism in Rats

We deleted the number of compounds with a relative peak area coefficient of variation of 30% or less in the quality control sample; 1320 metabolites (positive ion mode) and 460 metabolites (negative ion mode) with identification information were reserved.

#### 3.9.1. Screening for Differential Metabolites

**Figure 8:**
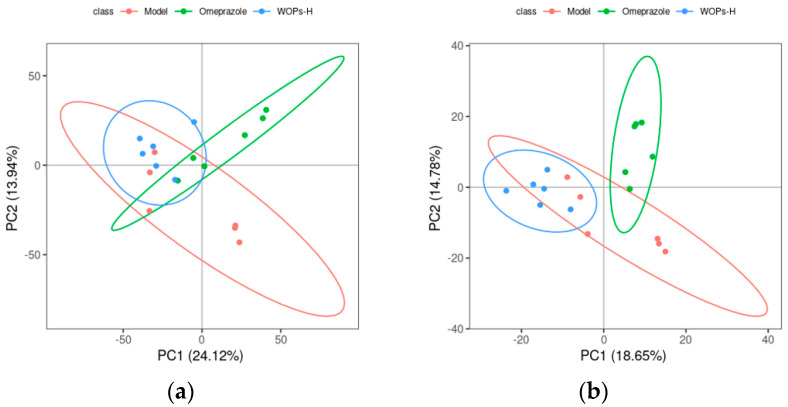
Score chart of PCA analysis model. (**a**) Positive ion mode; (**b**) negative ion mode. PC1 represents the first principal component, PC2 refers to the second principal component, and the number in parentheses is the score of the principal component, which represents the percentage of the overall variance explained for the specific principal component. The ellipses are 95% confidence intervals. Each point represents one sample, with six samples per group, and different groups are labeled with different colors.

As shown in Figure 8, the separation between the model, omeprazole, and WOPs-HG groups was not significant in the PCA score plot. A further analysis by PLS-DA revealed that the values of R2Y(cum) were 1 or close to 1, Q2(cum) were higher than 0.5, and Q2 was less than 0. This demonstrated that the model was stable and reliable, and the model had a good prediction effect (Table 2).

**Figure 9:**
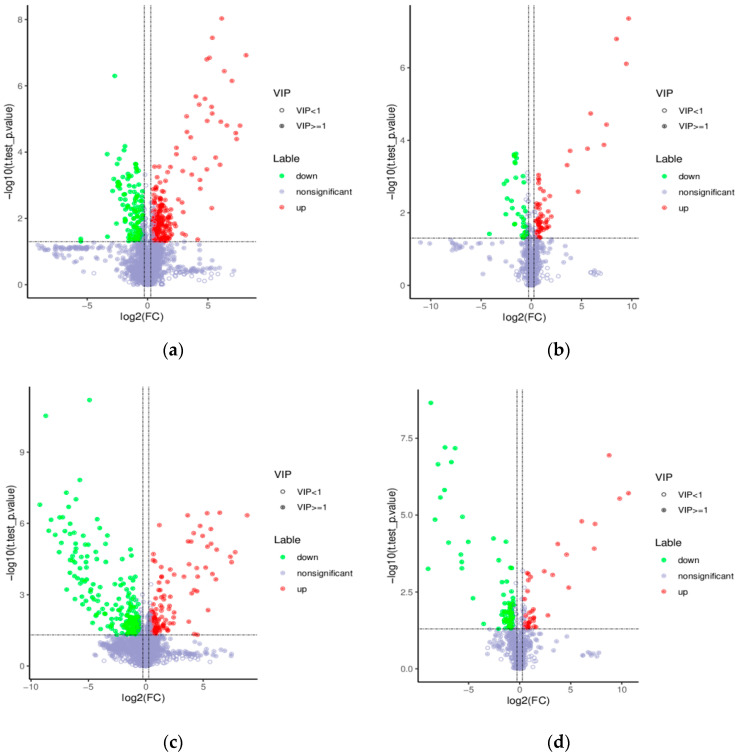
Volcano map of differential metabolites. (**a**) Positive ion mode (WOPs-HG group vs. model group); (**b**) negative ion mode (WOPs-HG group vs. model group); (**c**) positive ion mode (WOPs-HG group vs. omeprazole group); (**d**) negative ion mode (WOPs-HG group vs. omeprazole group). Green is the downregulated differential metabolite (labeled green), red is the upregulated differential metabolite (labeled red), and metabolites without difference are labeled with purple-gray.

Based on the PLS-DA, the further analyzed results of the fold changes and *t*-tests were adopted to screen for differential metabolites, according to the conditions described in Section 2.9. The volcano map of the screened differential metabolites is presented in Figure 9. There were 357 differential metabolites in the positive ion mode and 99 differential metabolites in the negative ion mode between the WOPs-HG group and the model group. Meanwhile, there were 373 differential metabolites in the positive ion mode and 120 differential metabolites in the negative ion mode between the WOPs-HG group and the omeprazole group.

#### 3.9.2. Differential Metabolite Analysis

**Figure 10:**
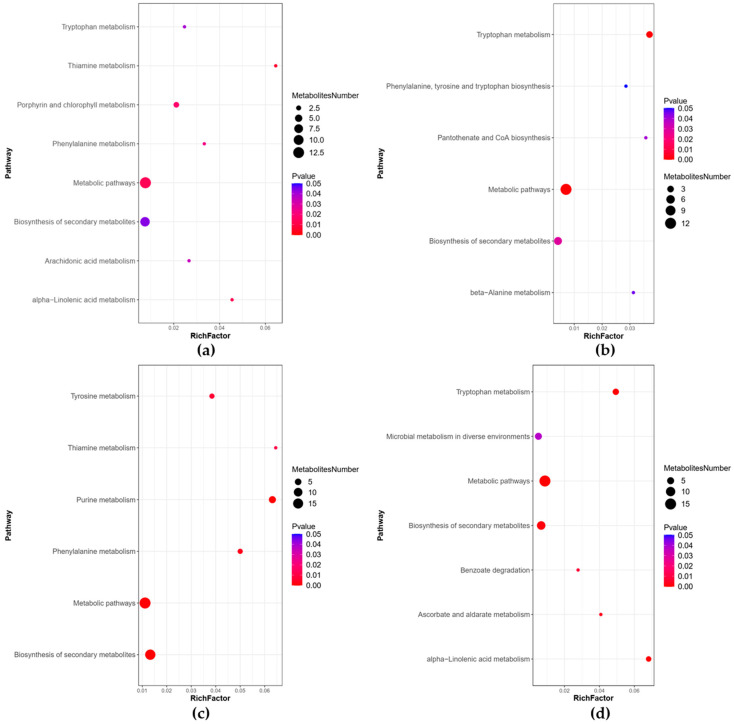
Bubble plots for metabolic pathway enrichment analysis. (**a**) Positive ion mode (WOPs-HG group vs. model group); (**b**) negative ion mode (WOPs-HG group vs. model group); (**c**) positive ion mode (WOPs-HG group vs. omeprazole group); (**d**) negative ion mode (WOPs-HG group vs. omeprazole group). *X*-axis enrichment factor (RichFactor) is the number of differential metabolites annotated to the pathway divided by all identified metabolites annotated on the pathway. The larger the value, the greater the proportion of differential metabolites. The dot size represents the number of differential metabolites annotated to this pathway.

The results of the enrichment analysis of the KEGG database revealed eleven pathways that were significantly enriched between the WOPs-HG group and the model group (Figure 10a,b), including the tryptophan metabolism, pantothenate and CoA biosynthesis, beta-Alanine metabolism, phenylalanine, tyrosine and tryptophan biosynthesis, thiamine metabolism, alpha-Linolenic acid metabolism, porphyrin and chlorophyll metabolism, phenylalanine metabolism, arachidonic acid metabolism, metabolic pathways, and biosynthesis of secondary metabolites. Eleven pathways were also significantly enriched between the WOPs-HG group and the omeprazole group (Figure 10c,d), including the metabolic pathways, tryptophan metabolism, alpha-Linolenic acid metabolism, ascorbate and aldarate metabolism, benzoate degradation, microbial metabolism in diverse environments, purine metabolism, biosynthesis of secondary metabolites, phenylalanine, and tyrosine and thiamine metabolism.

## 4. Discussion

Indomethacin is commonly applied in the clinical therapy of inflammatory diseases such as rheumatoid arthritis and osteoarthritis, but it has been reported to induce gastrointestinal side effects in both rats and humans [26]. In this study, indomethacin was chosen to induce gastric ulcers in rats at a dose of 48 mg/kg, and the maximum injury was observed 1 h after the indomethacin injection. Based on the United States Food and Drug Administration recommendation, this dose in rats would be considered a high dose when converted to the human equivalent through normalization to the body surface area (BSA). This is about 7.78 mg/kg, which equates to a 467 mg dose of indomethacin for a 60 kg person [27].

Most nonselective NSAIDs are weakly acidic, relatively nonionized, and lipophilic in gastric juice, which can cause cytotoxic effects, leading to cell death and destroying the integrity of the epithelial cell layer [28]. In this study, the rats were treated intraperitoneally with 48mg/kg indomethacin for 1 h, and it was found that an indomethacin treatment-induced severe gastric mucosa injury exhibited linear and cord-like bleeding bands, and caused an increase in the ulcer index in the gastric tissues of rats in the model group, whereas the omeprazole and WOPs pretreatment ensured less gastric lesions and a significant reduction in the ulcer indices. In addition, no significant difference in the GUI was observed between the WOPs 0.88 g/kg group and the omeprazole group (Figure 1). This result shows that WOPs supplementation, especially at a high dose of 0.88 g/kg, could protect from gastric ulceration in rats challenged with indomethacin.

The deleterious action of NSAIDs is largely due to decreased prostaglandin (PGs) synthesis as a result of cyclooxygenase (COX) suppression [29]. PGs play critical roles in maintaining the integrity of the gastric mucosa and protecting it from damage. The cytoprotective actions of PGs were first reported by Andre Robert in 1979 [30]. Robert provided experimental evidence that PGs exhibit protective effects against gastric mucosal lesions induced by necrotizing substances, including ethanol, acids, concentrated bile, and even boiling water. The gastric protective effects of prostaglandins are mainly realized by the regulation of mucosal blood flow, gastric mucus and bicarbonate synthesis, epithelial cell proliferation, and the inhibition of the leukocyte aggregation and direct cytotoxic injury [4,31]. PGs mainly include PGA, PGF, PGI, and PGE. PGE2 is the most important of the PGs, with the highest content in the human gastrointestinal tract. In this study, the results indicated that the PGE2 levels in the gastric tissues of the rats in the model group were greatly decreased compared with the normal rats, suggesting that indomethacin induced the inactivation of prostaglandin synthase and thus decreased prostaglandin biosynthesis. However, the levels of PGE2 in the WOPs pretreatment groups (0.44 and 0.88 g/kg) were higher than those in the model and omeprazole groups. The result suggested that PGE2 is involved in the beneficial influence of WOPs on indomethacin-induced gastric mucosal damage.

NSAID-induced gastric ulcers also demonstrate enormous nitric oxide (NO) generation, leading to a pervasive elevated susceptibility to mucosal lesions [32]. The inducible nitric oxide synthase can release large amounts of NO when activated by harmful factors, inducing a vascular microcirculation disturbance and accelerating the formation of a gastric ulcer [33,34]. Additionally, neutrophil infiltration is a key process in the development of gastric mucosal lesions. NSAIDs can inhibit COX activity, promoting arachidonic acid metabolism under the action of lipoxygenase (LOX). It leads to the production of leukotriene B4, leukotriene C4, and leukotriene D4 in large quantities, causing neutrophil aggregation and infiltration, thus aggravating gastric mucosal injury [35]. Myeloperoxidase (MPO) determination has been widely used as an important parameter of neutrophil infiltration. In our study, pretreatment with WOPs significantly inhibited the elevation of the MPO and NO levels caused by indomethacin compared with the model group. In addition, the inhibitory influence of the WOPs 0.44 and 0.88 g/kg on the MPO level was higher than that of omeprazole. The results indicated that WOPs can protect against indomethacin-related gastric ulcers by increasing the synthesis of PGE2 and reducing the MPO and NO levels.

An NSAIDs-induced deleterious action was always accompanied by the excessive generation of oxygen free radicals and proinflammatory factors, such as superoxide radical anions, hydroxyl radicals, tumor necrosis factor α (TNF-α), and interleukin (IL)-1β (IL-1β) [36]. These mechanisms, in combination with those linked to PG inhibition, influence the pathogenesis of gastric mucosal injury associated with gastrointestinal damage caused by NSAIDs [37]. Oxidative stress participates in the development of an NSAIDs-related gastric ulcer via a prostaglandin-independent mechanism [5]. In particular, indomethacin causes gastric injury by decreasing the content of antioxidants (e.g., SOD and GSH) and increasing lipid peroxidation products (e.g., MDA) [38]. SOD and GSH are important enzymes that scavenge superoxide anion radicals and protect cells from oxidative damage [39]. MDA cross-links DNA and proteins, disturbs cell division, and destroys the structure and function of proteins and enzymes [40]. Our study demonstrated that treatment with 48mg/kg indomethacin remarkably decreased the levels of SOD and GSH, while it elevated the levels of TNF-α, IL-17A, IL-1β, and MDA in the model group rats. However, WOPs pretreatment could greatly decrease those antioxidants and elevate the inflammatory cytokines. According to these results, WOPs displayed an obvious beneficial effect of an indomethacin-induced oxidative stress imbalance and excessive inflammatory reaction of gastric mucosa.

COX is a rate-limiting enzyme in the eicosanoids synthesis. Its two isoforms, COX-1 and COX-2, are critical enzymes in the metabolism of arachidonic acid to the intermediate prostaglandins [41]. COX-1 exists in most tissues and its expression is basically constant. It is mainly responsible for the synthesis of PGs, which maintain gastric integrity and promote ulcer healing. The inhibition of COX-1 activity can decrease PGs synthesis in gastric mucosa, lead to the reduction in gastric mucosal defiance function, and cause gastric mucosal erosion, ulcer, and other injuries [18]. COX-2 is an inducible rate-limiting enzyme. Its expression is commonly low under basal conditions, but it can be expressed after stimulation by pathological reactions [41,42]. Both COX-1 and COX-2 play a critical role in the synthesis of PGs. Standard NSAIDs have significant inhibitory effects on COX-1 and COX-2, such as indomethacin or ibuprofen [43]. Our project outcomes indicated that the mRNA expression of COX-1 and COX-2 was obviously downregulated in the model group compared to the normal group of rats. Notably, administration with omeprazole and WOPs remarkably upregulated the mRNA expression of COX-1 and COX-2 in comparison with that of the model group. The result suggested that WOPs had a positive effect on the prevention of indomethacin-induced gastric ulcers, which may be attributed to prompting the synthesis of the gastric mucosal protective factor PGE2 by increasing the COX-1 and COX-2 expression of gastric tissue. There are two main metabolic pathways of arachidonic acid (AA) in vivo: the synthesis of PGs, prostacyclin, and thromboxane A2 (TXA2) catalyzed by cyclooxygenase (COX) and the production of leukotriene B4 (LTB4), leukotriene C4 (LTC4), and leukotriene D4 (LTD4) catalyzed by lipoxygenase (LOX) [44]. The diagram of the metabolic pathway enriched by the KEGG metabolic pathway showed that WOPs could be involved in arachidonic acid metabolism by downregulating the production of LCT4. In addition, the metabolic results indicated that WOPs could upregulate the expression of tryptophan, phenylalanine, tyrosine, and alpha-Linolenic acid, which may be one of the mechanisms of its gastroprotective effect.

## 5. Conclusions

WOPs pretreatment could protect the gastric mucosa from indomethacin-induced injury. Its gastroprotective effects may be a result of its attenuation of the severity of the gastric mucosal damage caused by aggressive factors and its improvement in the morphological structure of gastric mucosa cells, its enhancement of the oxidative stress capacity, and its suppression of an excessive inflammatory reaction, as well as the lipid peroxidation marker altered by risk factors. WOPs could enhance the COX-1 and COX-2 expression of gastric tissues in rats, improving the synthesis of prostaglandins. Moreover, the metabolomics results indicated that WOPs are involved in the arachidonic acid, tryptophan, phenylalanine, tyrosine, and alpha-Linolenic acid metabolism, exerting a protective effect on gastric mucosa. This study reported the beneficial effects of WOPs on indomethacin-induced gastric ulcers and confirmed a better gastroprotective effect at a dose of 0.88 g/kg, providing an important perspective for the application of WOPs in gastric ulcers associated with NSAIDs. Further studies are needed to investigate the potential protective role of WOPs on ulcerative lesions occurring in the lower intestinal tract and determine the optimal supplemental dose of WOPs in humans and its clinical applications.

## Figures and Tables

**Table 1:** Effects of WOPs on body weight, food intake, and food utilization in rats.

Groups	Number	Body Weight (g)		Food Intake (G)	Food Utilization (%)
		Initial	Final		
Normal	10	221.00 ± 12.83	399.25 ± 18.21	650.00 ± 63.51	27.54 ± 4.34
Model	10	217.67 ± 8.23	387.33 ± 26.31	665.31 ± 49.19	25.74 ± 5.44
Omeprazole	10	210.58 ± 14.00	379.50 ± 41.84	651.00 ± 59.62	25.90 ± 6.77
WOPs 0.22 g/kg	10	214.78 ± 12.94	382.11 ± 27.54	665.25 ± 69.42	25.28 ± 3.62
WOPs 0.44 g/kg	10	212.62 ± 12.05	394.23 ± 37.72	681.19 ± 43.83	26.61 ± 4.57
WOPs 0.88 g/kg	10	215.73 ± 9.17	379.73 ± 32.63	678.84 ± 21.29	24.15 ± 4.79

WOPs—walnut oligopeptides.

**Table 2:** The PLS-DA model parameters.

Mode	Group	A	R2Y(cum)	Q2(cum)	R2	Q2
Positive	WOPs-H vs. Model	3	1	0.77	(0.0, 0.94)	(0.0, −0.74)
Positive	WOPs-H vs. Omeprazole	3	1	0.83	(0.0, 0.95)	(0.0, −0.90)
Negative	WOPs-H vs. Model	3	1	0.67	(0.0, 0.93)	(0.0, −0.77)
Negative	WOPs-H vs. Omeprazole	3	1	0.86	(0.0, 0.94)	(0.0, −0.99)

A means the principal component number; R2Y(cum) refers to the interpretation rate for Y matrix; Q2(cum) represents the predictive ability. R2 and Q2 are the intercepts of the Y axis of the regression line during permutation experiment.

## Data Availability

The data presented in this study are available on request from the corresponding author. The data are not publicly available due to privacy. The studies not involving humans.
